# Cluster analysis of karyotype similarity coefficients in *Epimedium* (Berberidaceae): insights in the systematics and evolution

**DOI:** 10.3897/phytokeys.161.51046

**Published:** 2020-09-15

**Authors:** Lin-Jiao Wang, Meng-Di Gao, Mao-Yin Sheng, Jie Yin

**Affiliations:** 1 Institute of Karst Research, Guizhou Normal University, Guiyang 550001, China Guizhou Normal University Guiyang China; 2 National Engineering Research Center for Karst Rocky Desertification Control, Guiyang 550001, China National Engineering Research Center for Karst Rocky Desertification Control Guiyang China; 3 Guizhou Engineering Laboratory for Karst Rocky Desertification Control and Derivative Industry, Guiyang, Guizhou 550001, China Guizhou Engineering Laboratory for Karst Rocky Desertification Control and Derivative Industry Guiyang China

**Keywords:** Cluster analysis, cytogenetics, Ranunculales, similarity coefficient, systematics, *
Vancouveria
*

## Abstract

In order to evaluate the genome evolution and systematics, karyotype analysis of mitotic metaphase chromosomes in 51 taxa of *Epimedium* and two species of *Vancouveria* was conducted. The 53 taxa were clustered, based on their karyotype similarity coefficients. Results showed that the 53 taxa studied were all diploid with 12 chromosomes (2*n* = 2*x* = 12). Each taxon had one pair of satellites located on pair I of homologous chromosomes. Moreover, the karyotype types of the 53 taxa studied were all type 1A or 2A of Stebbins. It can be concluded that the karyotypes between species are indeed very similar and the genome of *Epimedium* was conservative in evolution. The cluster analysis of karyotype similarity coefficients could provide valuable clues for the systematics and taxonomy of *Epimedium*. Results of the cluster analysis strongly supported the previous taxonomic division of E. subg. Rhizophyllum and E. subg. Epimedium. The results also showed that the interspecific relationship was closely correlated with geographical distribution in E. subg. Epimedium and the taxa native to east Asia had the highest genetic diversity in *Epimedium*. Finally, the origin of the modern geographical distribution of *Epimedium* was inferred. Results of the present study have significant scientific values in further studies on resource utilisation, taxonomy and phylogeny in *Epimedium*.

## Introduction

Barrenwort, (*Epimedium* L., Berberidaceae), is an important traditional medicinal plant in China. It is effective in strengthening kidney and curing rheumatism (Guo et al. 2019), widely used in the treatment of osteoporosis, hypertension and coronary heart disease and also used to strengthen immunity and prevent dementia (Guo et al. 2008). Recent studies have verified that *Epimedium* can inhibit the growth of cancer cells *in vitro* (Wang et al. 2015). Moreover, *Epimedium* has become very popular in horticulture due to its excellent characteristics, such as exotic flower shape, variable flower colour, perennial evergreen plant etc. In recent years, studies on *Epimedium* have become considerably more common (Xu et al. 2019; Qu et al. 2020).

Currently, over 60 species of *Epimedium* are globally recognised (Qu et al. 2020). China is the main distribution centre, with more than 50 species and six varieties reported (Stearn 2002). The genus ranges from Japan in east Asia to Algeria in north Africa (Ying 2002), with the distribution mainly divided into two regions: 1) the Mediterranean and West Asia; and 2) East Asia. As such, the genus is typical from temperate areas (Ying 2002). For a long time, *Epimedium* has been neglected in systematics and taxonomy works due to its numerous species of difficult circumscription (Guo et al. 2008; Sheng and Wang 2010; Qu et al. 2020).

Stearn (2002) provided the most comprehensive taxonomic account of *Epimedium*, based on the geographical distribution, number of leaves on the stem, flower size, petal shape, relative size between petal and sepal and chromosomal C-banding. In the taxonomic system of Stearn (2002), *Epimedium* was divided into two subgenera: E. subg. Epimedium and E. subg. Rhizophyllum. Epimedium subg. Epimedium was divided into four sections: E. sect. Diphyllon (native to China), E. sect. Macroceras (native to Japan, Korea, north-eastern China and the far east of Russia), E. sect. Polyphyllon (native to West Himalaya) and E. sect. Epimedium (native to Europe, Caucasus and northern Turkey). Epimedium sect. Diphyllon (native to China), the most complex group in the genus, was further divided into four series: E. ser. Campanulatae, E. ser. Davidianae, E. ser. Dolichocerae and E. ser. Brachycerae.

Although Stearn’s (2002) taxonomic system is very comprehensive and the distinction between categories is clear, urgent problems remain unsolved regarding the taxonomy and systematics of *Epimedium*. For example: 1) the interspecific relationships are still unclear, especially in E. sect. Diphyllon (native to China); and 2) China is the diversity centre of this genus, but the process of diversification is still unclear, especially regarding the modern discontinuous distribution (Nakai et al. 1996; Sun et al. 2004; 2014; Guo et al. 2019; Sheng and Wang 2010).

Karyotype analysis has been extensively used in plant taxonomy and systematics. It can provide significant cytological data for the studies on origin, evolution and interspecific relationship in plants (Sheng and Chen 2007a). Since the 1980s, many cytological works have been conducted in *Epimedium*. Krikorian et al. (1983) reported the karyotypes of 11 *Epimedium* species native to Japan. Tanaka and Takahashi (1981) and Takahashi (1989) reported the chromosome C-banding of 26 *Epimedium* taxa, successively. The comparative study on karyotypes of 18 *Epimedium* species was carried out by Sheng and Chen (2007a, b). These results showed that all *Epimedium* were diploids with 12 chromosomes (2*n* = 2*x* = 12). All the karyotypes were symmetric to Stebbins’ type 2A or 1A and similar to each other with one pair of middle satellite chromosomes. It can be seen that differences between homologous chromosomes were insufficient and karyotypes between species were very similar. Despite many relevant studies on the karyology of *Epimedium* (Sheng et al. 2010; Yan et al. 2016; Zhang et al. 2018), all results further confirmed that the karyotypes are very similar and a traditional analysis could not provide more valuable cytological evidence for studies on taxonomy and systematics.

In the past 20 years, authors of the present study have collected 51 *Epimedium* and conducted their karyotype analysis of mitotic metaphase chromosomes in root tips. However, traditional karyotype analysis is very limited in studies focusing on the taxonomy and systematics of *Epimedium* since the karyotypes are very similar between the species. In the present study, the cluster analysis of karyotype similarity coefficients in the 53 taxa was conducted to try to provide cytological evidence for further studies on systematics and evolution of the genus.

## Materials and methods

### Materials studied

Fifty-three experimental taxa, including 51 *Epimedium* taxa and two *Vancouveria* species (the most closely-related genus) were collected from China, Japan, Germany and the United States. The details, including collection location and voucher specimens of these experimental materials, are detailed in Table 1. All the voucher specimens were conserved in the Institute of Karst Research, Guizhou Normal University. Parts of materials also were cultivated in a greenhouse of the Institute.

**Table 1.** The location and vouchers of the material studied.

No.	Taxon	location	Voucher
*** Epimedium ***			
subgenus Epimedium			
Section Diphyllon (native to China)			
Series *Campanulatae*			
1	*E. ecalcaratum* G.Y.Zhong	Baoxing, Sichuan, China	2008060210BX
2	*E. shuichengense* S.Z.He	Liupanshui, Guizhou, China	2009022501LPS
3	*E. platypetalum* K.Meyer	Hanzhong, Shanxi, China	2016030201HZ
Series *Davidianae*			
4	*E. davidii* Franch.	Kunming, Yunnan, China	2007070301 KM
5	*E. pauciflorum* K.C.Yen	Maoxian, Sichuan, China	2008060210MX
6	*E. flavum* Stearn	Tianquan, Sichuan, China	2014050902TQ
7	*E. ilicifolium* Stearn	Zhenping, Shanxi, China	2015040501ZP
8	*E. mikinorii* Stearn	Enshi, Hubei, China	2016030102ES
Series *Dolichocerae*			
9	*E. membranaceum* K.Meyer	Shunso Garden, Japan	2015032005SH
10	*E. lishihchenii* Stearn	Yishan, Tongren, Guizhou, China	2008030201YS
11	*E. acuminatum* Franch.	Kaiyang, Guizhou, China	2004050101KY
12	*E. wushanense* T.S.Ying	Kaili, Guizhou, China	2007030201KL
13	*E. leptorrhizum* Stearn	Wuchuan, Guizhou, China	2009020101WC
14	*E. baojingense* Q.L.Chen & B.M.Yang	Baojing, Hunan, China	2008100601BJ
15	*E. chlorandrum* Stearn	Baoxing, Sichuan, China	2008060208BX
16	*E. luodianense* M.Y.Sheng	Luodian, Guizhou, China	2007050505LD
17	*E. pudingense* S.Z.He	Kaiyang, Guizhou, China	2007110405KY
18	*E. glandulosopilosum* H.R.Liang	Wushan, Chongqing, China	2014050201WS
19	*E. pseudowushanense* B.L.Guo	Leishan, Guizhou, China	2014050601LS
20	*E. franchetii* Stearn	Badong, Hubei, China	2015030201BD
21	*E. enshiense* B.L.Guo & Hsiao	Enshi, Hubei, China	2016030101ES
22	*E. sutchuenense* Franch.	Zhuxi, Hubei, China	2016030201ZX
23	*E. zhushanense* K.F.Wu & S.X.Qian	Zhushan, Hubei, China	2016030302ZS
Series *Brachycerae*			
24	*E. pubescens* Maxim.	Mt. Qingcheng, Sichuan, China	2008030201QC
25	*E. sagittatum* (Sieb. & Zucc.) Maxim.	Guiyang Arboretum, Guizhou, China	2007052001GY
26	E. sagittatum var. glabratum T.S.Ying	Enshi, Hubei, China	2016030105ES
27	*E. dolichostemon* Stearn	Lichuan, Hubei, China	2008110502LH
28	*E. truncatum* H.R.Liang	Dayong, Hunan, China	2015041901DY
29	*E. brevicornu* Maxim.	Taibai, Shanxi, China	2015042001TB
30	*E. myrianthum* Stearn	Jiangkou, Guizhou, China	2016060301JK
31	*E. stellulatum* Stearn	Shiyan, Hubei, China	2015032602SY
32	*E. fargesii* Franch.	Chengkou, Chongqing, China	2015031503CK
33	*E. elachyphyllum* Stearn	Songtao, Guizhou, China	2013060201ST
Section Macroceras (native to Japan, Korea, northeastern China, and the far east of Russia)			
34	*E. koreanum* Nakai	Tonghua, Jilin, China	2012030201TH
35	*E. grandiflorum* Morr.	Kochi, Japan	2016062503KO
36	E. grandiflorum var. thunbergianum (Miq.) Nakai	Miyagi, Japan	2016092530MI
37	E. grandiflorum var. higoense T.Shimizu	Kumamoto, Japan	2015100102KU
38	E. grandiflorum var. coelestre (Nakai) T.Shimizu	Gunma, Japan	2016120201GU
39	*E. sempervirens* Nakai	Fukui, Japan	2016102320FU
40	E. sempervirens var. hypoglaucum (Makino) Ohwi	Ishikawa, Japan	2015100205IS
41	E. sempervirens var. multifoliolatum T.Shimizu	Nara, Japan	2016051002NA
42	*E. trifoliatobinatum* (Koidz.) Koidz	Kochi, Japan	2012050301KO
43	*E. diphyllum* (Morren et Decne.) Lodd.	Hiroshima, Japan	2011080602HI
44	*E. cremeum* Nakai & F.Maek	Iwate, Japan	2011060502IW
45	*E. kitamuranum* Yamanaka	Tokushima, Japan	2010080502TO
46	*E. setosum* Koidz	Okayama,, Japan	2010080508OK
Section Epimedium (native Europe, Caucasia, and northern Turkey)			
47	*E. alpinum* L.	Munich, Germany	2008121401MU
48	*E. pubigerum* (DC.) Morren & Decne.	Munich, Germany	2008121501MU
Subgenus Rhizophyllum (native to Caucasia and North Africa)			
49	E. pinnatum subsp. colchicum Boiss	Arnold Arboretum, USA	2013060501AR
50	*E. pinnatum* cv. “Elegans”		
51	*E. perralderianum* Coss.	Arnold Arboretum, USA	2013060508AR
**Genus *Vancouveria***			
52	*V. hexandra* (Hook.) Morren & Decne.	Arnold Arboretum, USA	2013060509AR
53	*V. chrysantha* Greene	Arnold Arboretum, USA	2013060510AR

### Preparation of chromosome spreads and karyotype parameters

The preparation method of chromosome slides referred to the method of Sheng and Chen (2007a). Chromosome slides obtained were examined and photographed by a tri-ocular microscope with a CCD camera (BX52-DP72, Olympus, Japan). At least ten well-spread metaphase plates of each experimental taxa were selected to calculate the karyotype parameters. Long arm (L), short arm (S), total chromosome length (TCL), relative length (%), arm ratio (L/S), arm index and location of the centromere were measured and calculated. RLR (the relative length ratio of the longest and shortest chromosome) and P (the proportion of chromosomes with arm ratio over 2:1) were also calculated. The classification of chromosome type was conducted according to Levan et al. (1964) and the karyotype type was determined by Stebbins’ (1971) criteria.

### Calculation and cluster analysis of karyotype similarity coefficients

The calculation formula of karyotype similarity coefficients referred to Tan and Wu (1993):

$\ddot{e}=\tilde{a}\times \beta$ (1)

${\tilde{a}}_{ij}=\frac{\sum_{k=1}^{n}x_{ik}\cdot x_{jk}-\left(\sum_{k=1}^{n}x_{ik}\right)\left(\sum_{k=1}^{n}x_{jk}\right)}{\sqrt{\sum_{k=1}^{n}{\left(x_{ik}-\bar{x}\right)}^{2}}\cdot \sum {\left(x_{jk}-{\bar{x}}_{j}\right)}^{2}}$ (2)

$\beta =1-\frac{d}{D}$ (3)

In formula (3), *D* is the sum of distances and *d* is the square root of the product of inner distance (*d_i_*) and outer distance (*d_n_*):

$D=\sum_{k=1}^{n}\left\lfloor x_{ik}\right\rfloor +\sum_{k=1}^{n}\left\lfloor x_{jk}\right\rfloor$ (4)

$d=\sqrt{d_{i}\cdot d_{n}}$ (5)

$d_{i}=\sum_{k=1}^{n}\left|x_{ik}-x_{jk}\right|$ (6)

$d_{n}=\left|\sum_{k=1}^{n}\left|x_{ik}\right|-\sum_{k=1}^{n}\left|x_{jk}\right|\right|$ (7)

According to the above formulae, the karyotype similarity coefficients of mitotic metaphase chromosomes of all the 53 experimental taxa were calculated. Furthermore, the 53 experimental taxa were clustered, based on the karyotype similarity coefficients by the UPGMA method (Tan and Wu 1993). The cluster analysis was conducted and the dendrogram was drawn by the NTSYS-pc software (Version 2.10e).

## Results

### General characteristics of karyotype

**Figure 1.**
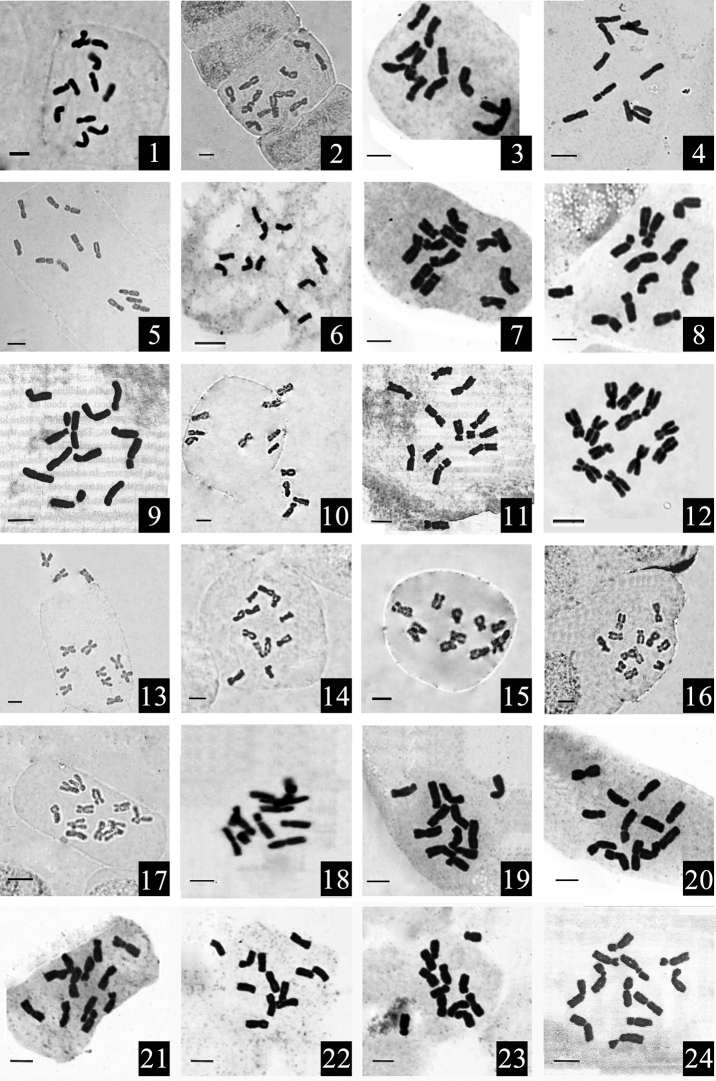
Mitotic metaphase chromosomes in 24 *Epimedium* species. **1***E. ecalcaratum***2***E. shuichengense***3***E. platypetalum***4***E. davidii***5***E. pauciflorum***6***E. flavum***7***E. ilicifolium***8***E. mikinorii***9***E. membranaceum***10***E. lishihchenii***11***E. acuminatum***12***E. wushanense***13***E. leptorrhizum***14***E. baojingense***15***E. chlorandrum***16***E. luodianense***17***E. pudingense***18***E. glandulosopilosum***19***E. pseudowushanense***20***E. franchetii***21***E. enshiense***22***E. sutchuenense***23***E. zhushanense***24***E. pubescens*. Scale bars: 5 μm.

**Figure 2.**
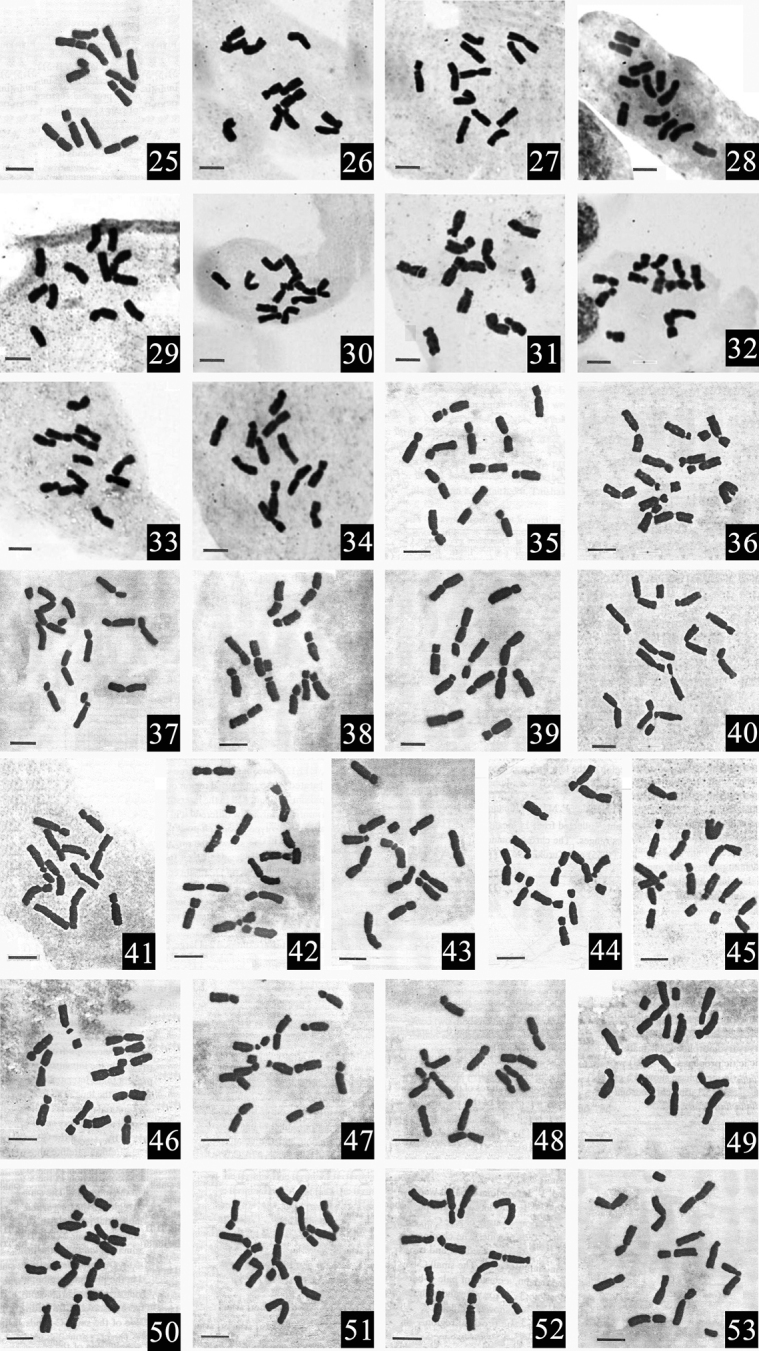
Mitotic metaphase chromosomes in 27 *Epimedium* taxa and two *Vancouveria* species. **25***E. sagittatum***26**E. sagittatum var. glabratum**27***E. dolichostemon***28***E. truncatum***29***E. brevicornu***30***E. myrianthum***31***E. stellulatum***32***E. fargesii***33***E. elachyphyllum***34***E. koreanum***35**E. grandiflorum var. grandiflorum**36**E. grandiflorum var. thunbergianum**37**E. grandiflorum var. higoense**38**E. grandiflorum var. coelestre**39***E. sempervirens***40**E. sempervirens var. hypoglaucum**41**E. sempervirens var. multifoliolatum**42***E. trifoliatobinatum***43***E. diphyllum***44***E. cremeum***45***E. kitamuranum***46***E. setosum***47***E. alpinum***48***E. pubigerum***49**E. pinnatum subsp. colchicum**50***E. pinnatum* cv. “Elegans” **51***E. perralderianum***52***V. hexandra***53***V. chrysantha*. Scale bars: 5 μm.

All 51 studied *Epimedium* taxa are diploid with 12 chromosomes (2*n* = 2*x* = 12). Pair I of homologous chromosomes in each studied taxon has one pair of satellites. The mitotic metaphase chromosomes of the 51 *Epimedium* taxa studied were illustrated in Fig. 1 (Material 1–24) and Fig. 2 (Material 25–51), respectively. The karyotype parameters of chromosome length, arm ratio, centromere index, RLR, P and karyotype are all detailed in Table 2. The average lengths of the six pairs of homologous chromosomes were 9.82, 9.04, 8.59, 8.02, 7.56 and 6.98 μm, respectively. The average arm ratios of the six pairs of homologous chromosomes were 1.28, 1.31, 1.44, 1.98, 2.12 and 2.06, respectively. In all 51 *Epimedium* taxa studied, the majority of chromosomes were metacentric (m) chromosomes or submetacentric (sm) chromosomes. Subtelocentric (st) chromosomes were few and telocentric (t) chromosomes were not found. The average values of RLR and P of the 51 taxa studied were 1.41 and 0.33, respectively. According to Stebbins’ (1971) classification, the karyotypes of all *Epimedium* taxa tested were highly symmetrical with type 2A or 1A. It also can be seen that the karyotypes between species were very similar in *Epimedium* species.

The two *Vancouveria* species studied also were diploid with 12 chromosomes (2*n* = 2*x* = 12). Furthermore, pair I of homologous chromosomes of each species also has one pair of satellites located. The mitotic metaphase chromosomes and the karyotype parameters of the two species studied are given in Fig. 2 (Material 52, 53) and Table 2 (Material 52, 53), respectively. The average lengths of the six pairs of homologous chromosomes were 9.65, 9.11, 8.69, 8.11, 7.30 and 7.16 μm, respectively. Furthermore, the average arm ratios of the six pairs of homologous chromosomes were 1.17, 1.10, 1.19, 1.43, 1.93 and 1.59, respectively. In the two *Vancouveria* species studied, only two chromosomal types, i.e. m and sm types, were found. The average RLR of the two species studied was 1.41. The P values of the two species studied were 0.00 and 0.17, respectively. Moreover, the karyotypes of the two species studied were 1A and 2A, respectively. It also can be seen that the karyotypes of the two *Vancouveria* species were very similar.

**Table 2.** Karyomorphological features of mitotic metaphase chromosomes in 51 *Epimedium* taxa and two *Vancouveria* species.

Materials No.	Chromosome length (μm)						Arm ratio						Centromeric index						RLR	P	KC
	1	2	3	4	5	6	1	2	3	4	5	6	1	2	3	4	5	6			
1	9.39	9.03	8.53	7.93	7.69	7.44	1.75	1.55	2.18	2.61	1.17	2.21	36.36	39.19	31.43	27.69	46.03	31.15	1.26	0.50	2A
2	9.79	8.87	8.53	8.52	8.07	6.22	2.15	1.20	1.64	1.55	2.33	1.84	31.76	45.45	37.84	39.19	30.00	35.19	1.57	0.33	2A
3	9.86	9.15	8.31	8.08	7.48	7.13	1.02	1.20	1.26	2.58	2.00	2.16	49.40	45.45	44.29	27.94	33.33	31.67	1.38	0.33	2A
4	9.40	9.29	9.18	7.52	7.30	7.30	1.30	1.40	1.68	2.58	2.14	1.75	43.53	41.67	37.35	27.94	31.82	36.36	1.29	0.33	2A
5	9.26	9.14	8.79	7.96	7.60	7.25	1.52	1.41	1.24	2.53	2.05	2.21	39.74	41.56	44.59	28.36	32.81	31.15	1.28	0.50	2A
6	10.22	8.93	8.30	7.98	7.45	7.12	1.82	1.63	3.11	1.27	2.68	2.72	35.42	38.10	24.36	44.00	27.14	26.87	1.44	0.50	2A
7	9.51	9.38	9.12	7.94	7.55	6.51	1.35	1.32	1.41	1.77	2.05	1.78	42.47	43.06	41.43	36.07	32.76	36.00	1.46	0.17	2A
8	9.49	9.39	8.22	7.83	7.63	7.44	1.31	1.13	1.63	2.20	2.71	3.00	43.30	46.88	38.10	31.25	26.92	25.00	1.28	0.50	2A
9	10.14	8.87	8.48	7.60	7.50	7.41	1.04	1.33	1.29	3.11	3.05	2.04	49.04	42.86	43.68	24.36	24.68	32.89	1.37	0.50	2A
10	9.76	8.70	8.47	8.35	7.53	7.18	1.59	1.55	1.32	2.23	1.56	2.21	38.55	39.19	43.06	30.99	39.06	31.15	1.36	0.33	2A
11	10.00	9.30	8.70	7.90	7.30	6.80	1.22	1.21	2.48	1.26	2.32	2.58	45.00	45.16	28.74	44.30	30.14	27.94	1.47	0.50	2A
12	9.90	8.79	8.30	8.02	7.55	7.42	1.05	1.22	1.39	1.71	2.21	2.16	48.75	45.07	41.79	36.92	31.15	31.67	1.33	0.33	2A
13	9.28	9.00	8.86	8.17	7.75	6.92	1.09	1.03	1.06	1.19	1.24	1.78	47.76	49.23	48.44	45.76	44.64	36.00	1.34	0.00	1A
14	9.75	8.86	8.48	7.85	7.72	7.34	1.41	1.41	2.05	1.30	3.07	1.76	41.56	41.43	32.84	43.55	24.59	36.21	1.33	0.33	2A
15	10.52	8.33	8.06	7.93	7.65	7.51	1.41	1.10	1.68	2.05	1.67	1.62	41.56	47.54	37.29	32.76	37.50	38.18	1.40	0.17	2A
16	10.83	8.46	8.31	8.30	8.01	6.08	1.28	2.17	1.67	1.43	1.84	2.42	43.84	31.58	37.50	41.07	35.19	29.27	1.78	0.33	2A
17	9.39	8.68	8.43	8.31	7.59	7.58	1.69	1.06	1.26	2.14	2.00	1.86	37.18	48.61	44.29	31.88	33.33	34.92	1.24	0.17	2A
18	9.68	9.45	9.22	8.43	7.52	5.70	1.50	1.68	1.79	1.96	2.14	1.17	40.00	37.35	35.80	33.78	31.82	46.00	1.70	0.17	2A
19	9.28	9.27	8.82	8.70	6.96	6.95	1.29	1.05	2.04	1.78	2.16	2.33	43.75	48.75	32.89	36.00	31.67	30.00	1.33	0.50	2A
20	10.43	9.01	8.75	7.82	7.11	6.87	1.15	1.17	1.00	2.14	2.16	2.41	46.59	46.05	50.00	31.82	31.67	29.31	1.52	0.50	2A
21	9.23	8.78	8.43	8.42	7.74	7.39	1.05	2.04	1.28	1.35	1.68	2.37	48.75	32.89	43.84	42.47	37.31	29.69	1.25	0.33	2A
22	9.41	8.77	8.76	8.66	7.36	7.03	1.23	1.31	1.53	1.29	1.34	1.95	44.83	43.21	39.51	43.75	42.65	33.85	1.34	0.00	1A
23	10.73	8.45	8.32	7.91	7.90	6.70	1.05	1.86	1.00	2.47	1.81	1.78	48.75	34.92	50.00	28.81	35.39	36.00	1.60	0.17	2A
24	9.50	9.23	8.40	8.21	8.12	6.55	1.10	1.56	2.14	1.34	2.14	2.23	47.57	39.00	31.87	42.70	31.82	30.99	1.45	0.50	2A
25	10.04	8.61	8.41	8.40	7.75	6.79	1.06	1.31	1.05	2.52	1.89	2.38	48.57	43.33	48.86	28.41	34.57	29.58	1.48	0.33	2A
26	9.18	8.99	8.98	8.42	7.56	6.88	1.46	1.19	1.09	1.10	1.82	1.32	40.63	45.74	47.87	47.73	35.44	43.06	1.33	0.00	1A
27	10.46	9.76	8.35	7.64	6.94	6.84	1.00	1.16	1.02	1.17	2.14	1.06	50.00	46.39	49.40	46.05	31.88	48.53	1.53	0.17	2A
28	9.97	9.47	8.85	7.85	7.61	6.23	1.05	1.17	1.15	1.25	2.21	1.78	48.75	46.05	46.48	44.44	31.15	36.00	1.60	0.17	2A
29	9.75	9.26	8.48	7.70	7.61	7.22	1.22	1.44	2.00	1.55	1.36	1.24	45.00	41.05	33.33	39.24	42.31	44.59	1.35	0.00	1A
30	9.04	9.03	8.48	8.47	7.49	7.48	1.00	1.65	1.20	2.50	1.96	2.24	50.00	37.80	45.45	28.57	33.82	30.88	1.21	0.33	2A
31	9.84	9.11	8.93	7.58	7.31	7.22	1.27	1.06	1.06	2.36	2.52	1.76	44.04	48.51	48.48	29.76	28.40	36.25	1.36	0.33	2A
32	10.31	8.68	8.67	8.66	7.38	6.32	2.03	1.31	1.11	1.96	1.86	1.84	32.95	43.24	47.30	33.78	34.92	35.19	1.63	0.17	2A
33	9.03	8.59	8.48	8.37	8.26	7.27	1.41	1.69	1.41	1.17	1.88	1.75	41.46	37.18	41.56	46.05	34.67	36.36	1.24	0.00	1A
34	9.67	9.25	8.84	7.93	7.19	7.10	1.44	1.20	1.18	1.74	2.48	2.07	41.03	45.54	45.79	36.46	28.74	32.56	1.36	0.33	2A
35	9.57	9.01	8.78	7.84	7.83	6.96	1.33	1.24	1.09	1.68	2.54	2.03	42.98	44.74	47.75	37.37	28.28	32.95	1.38	0.33	2A
36	10.18	9.19	8.12	7.96	7.58	6.97	1.18	1.07	2.03	1.12	2.54	2.14	45.86	48.33	33.02	47.12	28.28	31.87	1.46	0.50	2A
37	9.85	9.16	8.70	7.63	7.56	7.10	1.22	1.35	1.19	1.86	2.54	2.00	44.96	42.50	45.61	35.00	28.28	33.33	1.39	0.17	2A
38	9.53	9.36	8.86	7.69	7.36	7.19	1.15	1.20	1.36	1.97	2.52	2.44	46.49	45.54	42.54	33.70	28.41	29.10	1.33	0.33	2A
39	10.09	8.74	8.67	7.78	7.77	6.95	1.01	1.13	1.11	2.35	2.15	2.32	49.63	47.01	47.41	29.81	31.73	30.11	1.45	0.50	2A
40	9.75	9.04	8.54	7.75	7.74	7.17	1.09	1.07	1.16	2.27	2.09	2.45	47.79	48.41	46.22	30.56	32.41	29.00	1.36	0.50	2A
41	9.48	9.11	8.74	8.15	7.70	6.82	1.33	1.37	1.11	2.55	1.97	2.17	42.97	42.28	47.46	28.18	33.65	31.52	1.39	0.33	2A
42	9.71	9.57	9.14	7.50	7.35	6.71	1.16	1.13	1.17	2.28	2.32	2.24	46.32	47.01	46.09	30.48	30.10	30.85	1.45	0.50	2A
43	10.31	9.02	8.94	7.89	7.00	6.85	1.17	1.29	1.27	2.38	2.48	1.66	46.09	43.75	44.14	29.59	28.74	37.65	1.51	0.33	2A
44	10.52	9.10	8.22	8.05	7.89	6.20	1.11	1.22	2.29	1.08	1.97	2.50	47.33	45.13	30.39	48.00	33.67	28.57	1.70	0.33	2A
45	9.96	8.92	8.85	8.11	7.22	6.93	1.14	1.05	1.45	3.40	1.97	2.24	46.67	48.76	40.83	22.73	33.67	30.85	1.44	0.33	2A
46	9.66	9.34	8.14	7.99	7.67	7.19	1.28	1.13	1.08	3.00	2.31	2.21	43.80	47.01	48.04	25.00	30.21	31.11	1.34	0.50	2A
47	10.65	9.92	8.13	7.16	7.15	7.00	1.08	1.10	1.04	2.26	2.52	2.07	48.09	47.54	49.00	30.68	28.41	32.56	1.52	0.50	2A
48	9.68	9.27	8.46	8.13	7.64	6.83	1.29	1.07	1.04	2.45	2.76	2.36	43.70	48.25	49.04	29.00	26.60	29.76	1.42	0.50	2A
49	10.18	8.47	8.32	7.95	7.57	7.51	1.11	1.07	1.29	2.06	2.29	2.16	47.45	48.25	43.75	32.71	30.39	31.68	1.36	0.50	2A
50	9.60	8.88	8.80	8.08	7.60	7.04	1.03	1.27	1.29	2.48	1.97	2.03	49.17	44.14	43.64	28.71	33.68	32.95	1.36	0.33	2A
51	9.94	8.89	8.43	7.76	7.61	7.38	1.24	1.41	1.29	2.68	1.59	2.38	44.70	41.53	43.75	27.18	38.61	29.59	1.35	0.33	2A
52	9.47	9.10	8.95	8.28	7.17	7.01	1.31	1.07	1.31	1.64	1.82	1.69	43.31	48.36	43.33	37.84	35.42	37.23	1.35	0.00	1A
53	9.82	9.12	8.42	7.93	7.43	7.30	1.03	1.13	1.07	1.22	2.03	1.48	49.29	46.92	48.33	45.13	33.02	40.38	1.35	0.17	2A

Note: RLR, the relative length ratio of the longest and shortest chromosome; P, proportion of chromosomes with arm ratio over 2:1; KC, karyotype classification of Stebbins (1971).

### Cluster analysis of the karyotype similarity coefficients

**Figure 3.**
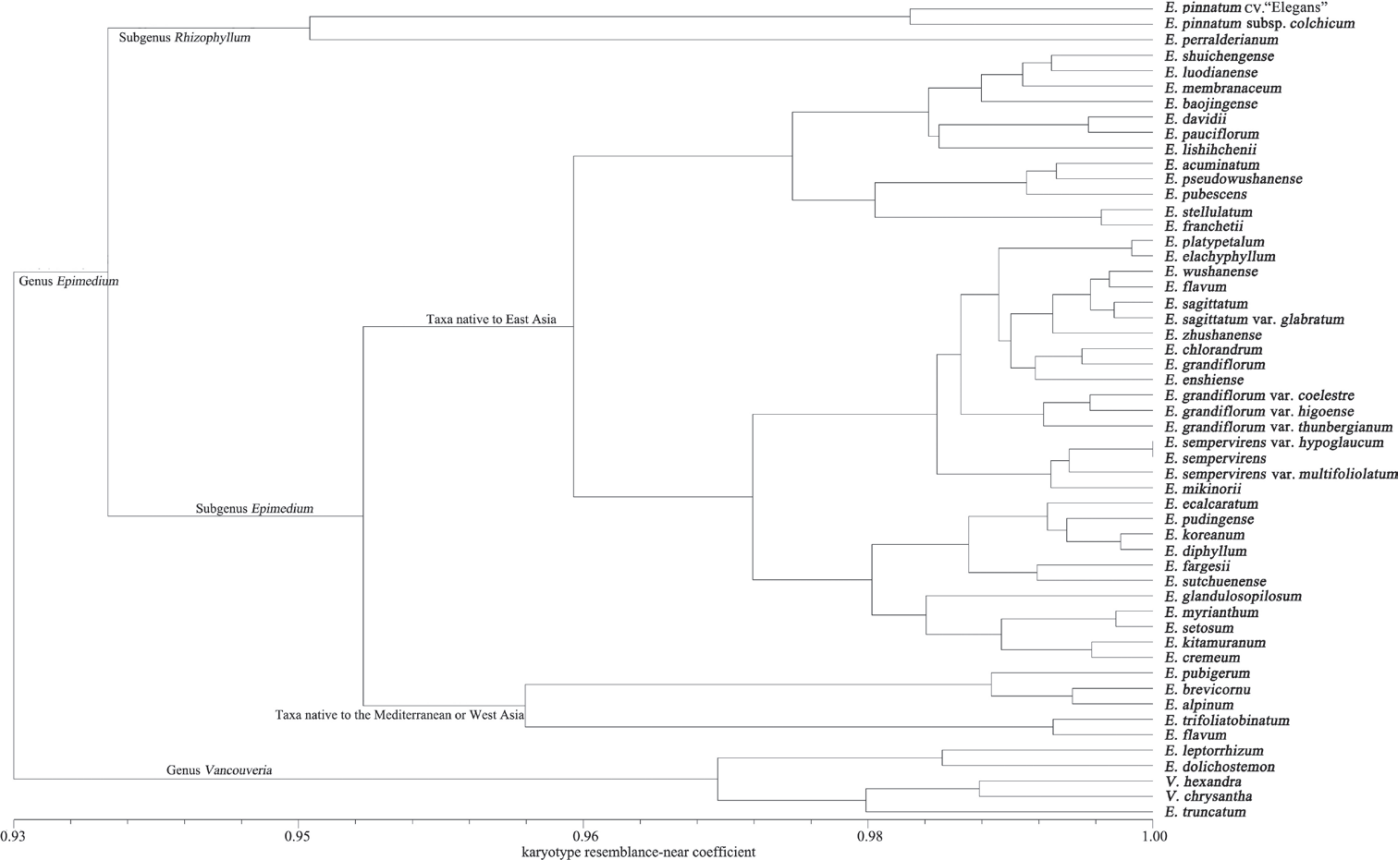
Diagram of cluster analysis of karyotype similarity coefficients in 51 *Epimedium* taxa and two *Vancouveria* species.

The karyotype similarity coefficients of the 53 taxa studied were calculated (Suppl. material 1: Table S1). The 53 taxa are clustered into two groups, mostly corresponding to the two genera (Fig. 3). However, some species of *Epimedium*, i.e. *E. truncatum*, *E. leptorrhizum* and *E. dolichostemon* were clustered into the group of genus *Vancouveria*.

Clustering results also showed *Epimedium*, firstly, split into two groups, E. subg. Epimedium and E. subg. Rhizophyllum. Epimedium subg. Epimedium is further split into two clusters, basically reflecting the geographical distribution, with one group mainly consisting of the taxa native to the Mediterranean and West Asia and the majority of the species of the other cluster native to east Asia. The clustering result also showed that the genetic diversity of *Epimedium* taxa native to east Asia was higher than those of Mediterranean and West Asian taxa. Finally, some species native to east Asia, i.e. *E. brevicornu*, *E. trifoliatobinatum* and *E. flavum*, were clustered into the group of the Mediterranean and West Asian taxa.

The karyotype similarity coefficients between the original species and its variant were significant, such as E. sempervirens var. sempervirens and E. sempervirens var. hypoglaucum, E. sempervirens var. multifoliolatum, E. sagittatum var. sagittatum and E. sagittatum var. glabratum and E. grandiflorum var. grandiflorum, E. grandiflorum var. thunbergianum, E. grandiflorum var. higoense and E. grandiflorum var. coelestre. It can be seen that the cluster analysis of karyotype similarity coefficients can provide reliable clues for studies on plant taxonomy and systematics, especially for those taxa with similar karyotypes between species and insufficiency differences between homologous chromosomes.

## Discussion and conclusion

### Relationships of *Epimedium* and related genera

Results of karyotype analysis showed that the genome of *Epimedium* was conservative in evolution and highly similar between species. The 51 *Epimedium* taxa tested are all diploid with the basic chromosomal number of 6 (2*n* = 2*x* = 12) and each of them has one pair of satellites located on pair I of homologous chromosomes. These results are consistent with the previous research reported (Tanaka and Takahashi 1981; Takahashi 1989; Yan et al. 2016; Zhang et al. 2018). Karyotypes of the 51 taxa studied are all highly symmetrical with the type 2A or 1A of Stebbins (1971). There has been the conclusion that species with symmetrical karyotypes usually are ancient and primitive in evolution and the karyotypes of evolutionary taxa are asymmetrical in spermatophytes (Stebbins 1971; Stace 2000). The highly symmetrical karyotypes in *Epimedium* species indicate that *Epimedium* should be conservative in evolution, consistent with other studies on the morphology (Stearn 2002; Xu et al. 2019), molecular biology (Kim and Jansen 1998; Kim et al. 2004), C-banding (Tanaka and Takahashi 1981; Takahashi 1989) and rDNA chromosomal location (Sheng and Wang 2010) in this genus. However, for a long time, studies on the systematics and taxonomy of *Epimedium* by using the traditional karyotype analysis have achieved little, because of the highly similar karyotypes between species and insufficient differences between homologous chromosomes (Guo et al. 2008).

*Epimedium* is phylogenetic related to *Vancouveria* (Tishler 1902; Berg 1972; Loconte and Estes 1989; Jin et al. 2018), being the species belonging to *Vancouveria* once classified into *Epimedium* (Stearn 1938). In the present study, results showed that the karyotypes of *Epimedium* and *Vancouveria* were highly similar. Karyotypes of the two *Vancouveria* species studies, with one pair of satellites located on pair I of homologous chromosomes, are very similar with some species of *Epimedium*. The two *Vancouveria* species, i.e. *V. hexandra* and *V. chrysantha*, were clustered into the group with *E. truncatum*, *E. leptorrhizum* and *E. dolichostemon* in the clustering of karyotype similarity coefficients. When compared with *Diphylleia* Michaux, *Dysosma* R. E. Woodson, *Podophyllum* L., and *Sinopodophyllum* Ying, the karyotype of *Epimedium* is significantly different and more symmetrical (Li 1986; Ma and Hu 1996), suggesting that the genus might be an ancient taxon in Berberidaceae and distantly related to these four genera. This conclusion also can be well supported by studies on morphology (Li et al. 2014), palynology (Zhang and Wang 1983; Wang et al. 2015), molecular markers (Wang et al. 2001; Sun et al. 2005; Zhang et al. 2014, 2016), isozymes (Sheng et al. 2011) and chemotaxonomy (Koga et al. 1991; Sheng et al. 2008). Therefore, it can be seen that karyotype analysis has important significance for studies on the relationships of *Epimedium* within Berberidaceae.

### Relationships of *Epimedium* infrageneric categories and species

Cluster analysis of karyotype similarity coefficients showed that, although the genome was conservative in evolution and the karyotypes between species were highly similar, the cluster analysis of karyotype similarity coefficients still provided some valuable clues for studies on phylogenetics and taxonomy in *Epimedium*. Clustering results of karyotype similarity coefficients strongly support the classification of the two subgenera of E. subg. Rhizophyllum and E. subg. Epimedium. Based on morphological characteristics and geographical distribution, E. Subg. Epimedium is clustered into two groups: 1) Mediterranean and Western Asian taxa and 2) East Asian taxa. The present results supported this classification, consistent with the previous studies on the morphology (Stearn 2002), cytology (Sheng et al. 2010; Zhang et al. 2018), molecular biology (Kim et al. 2004; Guo et al. 2018; Sajad et al. 2018) and phytochemistry (Guo et al. 2008), all of which confirmed the conclusion that the systematic relationship between species is closely related to the specific geographical distribution in this genus. The present results also showed that, although the vast majority of East Asia taxa could be clustered into one group, there are a few species clustered into other groups of *Epimedium* or the group of *Vancouveria*, indicating that the genetic diversity of East Asian taxa are the highest in the genus.

## Supplementary materials

Supplementary material 1 Table S1. Karyotype resemblance-near coefficients in 51 *Epimedium* taxa and two *Vancouveria* species Data type cytogenetic data https://binary.pensoft.net/file/452694This dataset is made available under the Open Database License (http://opendatacommons.org/licenses/odbl/1.0/). The Open Database License (ODbL) is a license agreement intended to allow users to freely share, modify, and use this Dataset while maintaining this same freedom for others, provided that the original source and author(s) are credited.Lin-Jiao Wang, Meng-Di Gao, Mao-Yin Sheng, Jie Yin
